# Clinical Characteristics, Risk Factors, and Outcomes of Portal Vein Thrombosis in Non-cirrhotic Patients: A Retrospective Study

**DOI:** 10.7759/cureus.109848

**Published:** 2026-05-28

**Authors:** Yahia Al-Hagawi, Nasser I Alqahtani, Saeed N Alsharif, Rafaat M Chakik, Sayed M Laeeq, Mohamed H Elgazzar, Saeed S Alqahtani, Mohammed N Alhajjaj, Salihah Y Al-Mani, Bushra M Asiri, Mohammad A Alqahtani, Abdulrahman A Alqahtani, Hatem A Ayied, Abdullah F Alahmari

**Affiliations:** 1 Gastroenterology, Armed Forces Hospital - Southern Region (AFHSR), Khamis Mushait, SAU; 2 Internal Medicine, Armed Forces Hospital - Southern Region (AFHSR), Khamis Mushait, SAU

**Keywords:** bowel ischemia, mortality prediction, portal vein thrombosis (pvt), risk factors, therapeutic anticoagulation

## Abstract

Non-cirrhotic portal vein thrombosis (PVT) is a rare but dangerous illness with few studies on risk factors and outcomes. This study sought to evaluate the clinical characteristics, risk factors, anticoagulation response, and consequences of non-cirrhotic PVT in hospitalized patients. We performed a retrospective study on 47 hospitalized patients with suspected non-cirrhotic PVT. Clinical, laboratory, and radiographic data were gathered. Descriptive statistics were employed to describe baseline attributes. The associations between risk factors and outcomes (thrombus resolution, intestinal ischemia, portal hypertension, and mortality) were examined using chi-square, Fisher's exact, Mann-Whitney U, and Kruskal-Wallis tests, if needed. Around 95.7% of patients (45/47) had radiologically verified non-cirrhotic PVT. The average age was 49.4 years (SD 19.5), and 61.7% were female. Obesity (BMI ≥30) was reported in 42.6%. Prothrombotic diseases were found in 17.0%, cancer in 25.5%, and a recent infection in 42.6%. Complete thrombus resolution occurred in 29.8% of cases, with no significant difference across anticoagulation methods (low molecular weight heparin (LMWH) 36.0%, direct oral anticoagulants (DOACs) 16.7%, and warfarin 50.0%; p=0.448). Bowel ischemia was substantially linked to recent infection (p* *= 0.016). The 90-day mortality rate was 17.0%. Significant univariate predictors of 90-day death were malignancy (p < 0.001), recent infection (p = 0.009), LMWH use (p = 0.018), and longer hospital stay (p = 0.041). There were no deaths among patients who received DOACs or warfarin. Non-cirrhotic PVT poses a significant mortality risk. Recent infection and underlying malignancy are strong indicators of negative outcomes. The link between anticoagulant type and mortality deserves additional exploration in prospective trials.

## Introduction

Portal vein thrombosis (PVT) is described as a thrombus that partially or completely occludes the portal vein. While PVT is typically seen in the presence of cirrhosis and portal hypertension, non-cirrhotic PVT is a unique clinical entity with its own etiology, natural history, and therapeutic considerations [1].

Non-cirrhotic PVT is a rare disorder, with an estimated yearly prevalence of 0.7 to 1.0 per 100,000 adults [2]. However, the true prevalence may be underestimated due to its frequently sluggish presentation and a lack of comprehensive screening in high-risk populations. Prothrombotic illnesses (e.g., factor V Leiden, myeloproliferative neoplasms), intra-abdominal infections, abdominal surgery, malignancy, and oral contraceptive use are also recognized risk factors [3].

Non-cirrhotic PVT might show clinically as asymptomatic incidental observations, acute abdominal pain, gastrointestinal bleeding from portal hypertension, or life-threatening bowel ischemia [4]. Anticoagulation is widely utilized to restrict thrombus growth and assist recanalization; however, the optimal anticoagulant regimen (low molecular weight heparin (LMWH), direct oral anticoagulants (DOACs), or vitamin K antagonists) is still debated [5]. Despite advances in imaging and anticoagulation, significant knowledge gaps exist regarding the predictors of thrombus clearance, the occurrence of sequelae such as intestinal ischemia and portal hypertension, and the risk factors for death in non-cirrhotic PVT. The majority of published studies are small, varied, or focused on specific patient populations [2, 6].

Thus, the current study was designed with the following objectives. Primary objectives were to determine the clinical characteristics and risk factors related to non-cirrhotic PVT and to analyze patient outcomes, including responsiveness to anticoagulant treatment. Secondary objectives included reporting the prevalence of non-cirrhotic PVT among hospitalized patients with suspected illness and examining consequences such as intestinal ischemia, portal hypertension, and mortality.

## Materials and methods

Study areas and settings

This study was carried out at the Armed Forces Hospital, Southern Region, a tertiary-care military hospital serving a vast catchment area in southern Saudi Arabia. The hospital offers a wide range of medical and surgical services, including a specialized hepatology and thrombosis center. The study was approved by the Research Ethics Committee of the Armed Forces Hospitals-Southern Region (approval no. H-06-KM-001).

Study design and period

This was a five-year retrospective cohort analysis of patients identified with non-cirrhotic PVT between January 1, 2020, and December 31, 2024. All eligible patients with first-time, imaging-confirmed non-cirrhotic PVT between 2020 and 2024 were identified using a systematic electronic medical record (EMR) query (the International Classification of Diseases, Tenth Revision (ICD-10) code I81, excluding cirrhosis) and human record review. The sample size (n=47) represents the entire cohort spanning five years at our center, corresponding with the known rarity of this illness (0.7-1.0 per 100,000 adults yearly).

Sampling population and sample size

Between January 1, 2020, and December 31, 2024, all adult patients (≥18 years) with a first-time, imaging-confirmed diagnosis of non-cirrhotic PVT were identified. Consecutive sampling was used. Using ICD-10 codes for PVT (code I81) in conjunction with the exclusion of cirrhosis codes (K74.6, K70.3, K71.7), all eligible patients were found by methodical inquiries via the hospital's EMR system. The sample size of 47 over five years is consistent with expected epidemiological rates and represents a complete rather than a sampled cohort, given the well-documented rarity of non-cirrhotic PVT. Medical records were then manually checked to confirm eligibility.

Inclusion and exclusion criteria

Patients were eligible if they met all of the following criteria: 18 years or older; had a confirmed diagnosis of non-cirrhotic PVT on cross-sectional imaging (CT, MRI, or Doppler ultrasound); and had complete medical records. Patients were excluded from the trial if they had cirrhosis or symptoms of chronic liver disease with portal hypertension before the PVT diagnosis or if their medical records were incomplete, missing important demographic, clinical, or outcome data.

Data collection tools and procedures

Patient information was retrieved retrospectively from the hospital's EMR system. A standardized data collection form (see Appendix A) was designed in advance and utilized to obtain pertinent information methodically. The following data domains were collected. Demographic characteristics were age (≤20, 20-40, 41-60, and >60 years); gender (male or female); BMI in kg/m², defined as healthy (18.5-24.9), overweight (25.0-29.9), or obese (≥30.0); and smoking history (yes or no). Prothrombotic disorders (such as factor V Leiden mutation, antiphospholipid syndrome, myeloproliferative neoplasms, protein C/S deficiency, and prothrombin gene mutation); malignancies (hepatobiliary, pancreatic, hematologic, or other solid tumors); recent infections (intra-abdominal infections or collections, sepsis, COVID-19, or both); recent surgery or trauma (abdominal surgery within four weeks before PVT diagnosis); and hormonal considerations.

The clinical presentation was classed as asymptomatic (incidental finding) or symptomatic, with symptoms such as abdominal pain (acute or chronic), gastrointestinal bleeding (hematemesis or melena), ascites, or a combination of these. Diagnostic findings included imaging modality (CT of the abdomen with or without contrast, MRI of the abdomen, or Doppler ultrasound) and thrombosis location. Treatment and outcomes included anticoagulation therapy type (LMWH, DOACs, or warfarin); thrombus resolution on follow-up imaging performed at least three months after anticoagulation initiation (complete, partial, uncertain, or no resolution); complications (bowel ischemia proven by imaging, surgery, or autopsy; portal hypertension evidenced by splenomegaly, esophageal or gastric varices, or ascites); length of hospital stay (LOS) in days; and mortality.

Data analysis

All statistical analyses were carried out using SPSS Statistics version 26.0 (IBM Corp., Armonk, NY, USA). All analyses were judged statistically significant with a two-tailed p-value of <0.05. The Shapiro-Wilk test was used to determine whether continuous variables were normal. Normally distributed variables were given as mean ± SD, while non-normally distributed variables were presented as median with interquartile range (IQR). Categorical variables were presented using frequencies and percentages (n, %).

For comparison of the categorical variables, Pearson's chi-square test (or Fisher's exact test for categorical variables with expected cell counts < 5) was used. For the continuous variables, an independent-samples t-test and a Mann-Whitney U test were used depending on whether the variables were normally or abnormally distributed. For comparisons of three or more groups, either the one-way ANOVA or the Kruskal-Wallis test was utilized.

A multivariable logistic regression was used to identify independent risk factors for non-cirrhotic PVT and 90-day death. Variables having a p-value < 0.10 in bivariate analysis were included in the multivariate model using the enter technique. The results were presented as adjusted odds ratios (aOR), with 95% confidence intervals (CI). Complete-case analysis was employed because there were no missing data for critical factors (age, gender, BMI, anticoagulation type, or mortality) among the 45 verified PVT patients. Given the limited sample size and descriptive nature of the analysis, no imputation was attempted for variables with minor missingness (for example, specific laboratory values that were not relevant to primary outcomes).

## Results

The study comprised 47 hospitalized patients with probable non-cirrhotic PVT. Except for age, all other variables were not normally distributed according to the Shapiro-Wilk results (p < 0.001). After radiological confirmation, 45 patients (95.7%) had verified PVT, whereas two (4.3%) had negative imaging and were eliminated from further investigation.

Table 1 summarizes the baseline characteristics of all 45 verified PVT patients. The average age was 49.4 years (SD 19.5, range 17-89 years), with 19 (40.4%) under the age of 40, 19 (27.7%) between the ages of 41 and 60, and 14 (29.8%) patients above the age of 60. Females represented 61.7% (n=29) of the cohort. Obesity (BMI ≥ 30 kg/m²) was observed in 42.6% (n=20), overweight in 29.8% (n=14), and healthy weight in 27.7% (n=13). The average BMI was 30.0 kg/m² (SD 7.7). Smoking was uncommon (n = 4, or 8.5%).

**Table 1:** Demographic and clinical characteristics of the study variables Except for age, all data is represented as a frequency in the form of n (%). Age is presented as mean±SD. Statistical significance is defined as a p-value < 0.05. PVT: Portal vein thrombosis;HTN: Hypertension; DM: Diabetes mellitus; MPN: Myeloproliferative neoplasm; MPV: Main portal vein; SMV: Superior mesenteric vein; LMWH: Low molecular weight heparin; DOACs: Direct oral anticoagulants; LOS: Length of hospital stay; IQR: Interquartile range

Variable		Measurement	Shapiro-Wilk (p-value)
Age (years) mean ± SD		49.4 ± 19.5	0.074
Age group (years)	≤20	1 (2.1%)	<0.001*
	20-40	19 (40.4%)	
	41-60	13 (27.7%)	
	>60	14 (29.8%)	
Gender	Male	18 (38.3%)	<0.001*
	Female	29 (61.7%)	
BMI (kg/m²) IQR		29 (18-54)	<0.001*
BMI group	Healthy weight: 14-24.9	13 (27.7%)	<0.001*
	Overweight: 25-29.9	14 (29.8%)	
	Obesity: 30 or higher	20 (42.6%)	
Smoking	No	43 (91.5%)	<0.001*
	Yes	4 (8.5%)	
Comorbidities	Nil	21 (44.7%)	<0.001*
	Hypertension (HTN)	5 (10.6%)	
	Diabetes mellitus (DM)	1 (2.1%)	
	Hypothyroidism	3 (6.4%)	
	Malignancy	2 (4.3%)	
	HTN and DM	6 (12.8%)	
	Mix and others	9 (19.1%)	
Prothrombotic	Nil	39 (83%)	<0.001*
	MPN	1 (2.1%)	
	Factor V Leiden	2 (4.3%)	
	Mix and others	5 (10.6%)	
Malignancy	Nil	35 (74.5%)	<0.001*
	Hematologic	3 (6.4%)	
	Hepatobiliary	6 (12.8%)	
	Pancreatic	3 (6.4%)	
Recent infection	Nil	27 (57.4%)	<0.001*
	Intra-abdominal infection or collection	16 (34%)	
	Sepsis	2 (4.3%)	
	Both	2 (4.3%)	
Abdominal surgery	No	22 (46.8%)	<0.001*
	Yes	25 (53.2%)	
Oral contraceptives/hormones	No	46 (97.9%)	<0.001*
	Yes	1 (2.1%)	
Clinical presentation	Nil	1 (2.1%)	<0.001*
	Abdominal pain	34 (72.3%)	
	Ascites	1 (2.1%)	
	Abdominal pain and ascites	5 (10.6%)	
	Abdominal pain and gastrointestinal (GI) bleeding	2 (4.3%)	
	Others	4 (8.5%)	
Imaging	CT abdominal	87.2%)	<0.001*
	MRI abdominal	5 (10.6%)	
	Doppler ultrasound	1 (2.1%)	
Thrombus location	Branches	14 (29.8%)	<0.001*
	Main portal vein (MPV)	36.2%)	
	MPV and branches	2 (4.3%)	
	MPV and superior mesenteric vein (SMV)	8 (17%)	
	No PVT	2 (4.3%)	
	Splenic vein	2 (4.3%)	
	Mix and others	2 (4.3%)	
Anticoagulation therapy	DOACs	18 (38.3%)	<0.001*
	LMWH	25 (53.2%)	
	Warfarin	3 (6.5%)	
Thrombus resolution	No	19 (40.4%)	<0.001*
	Yes	14 (29.8%)	
	Maybe	14 (29.8%)	
Complications	Nil	34 (72.3%)	<0.001*
	Portal (no splenomegaly, varices, or ascites)	8 (17%)	
	Bowel ischemia	5 (10.6%)	
LOS (days) IQR		7 (0-29)	<0.001*
30-day mortality	No	40 (85.1%)	<0.001*
	Yes	7 (14.9%)	
90-day mortality	No	39 (83%)	<0.001*
	Yes	8 (17%)	

Comorbidities included hypertension (HTN) (10.6%, n = 5), diabetes mellitus (DM (2.1%, n = 1), hypothyroidism (6.4%, n = 3), and mixed comorbidities (19.1%, n = 9). Prothrombotic disorders were found in 17.0% (n=8), including factor V Leiden (4.3%, n=2), myeloproliferative neoplasms (MPNs) (2.1%, n=1), and mixed prothrombotic states (10.6%, n=5). Malignancy was present in 25.5% (n=12), with the most prevalent kind being hepatobiliary (12.8%, n=6), followed by hematologic (6.4%, n=3) and pancreatic (6.4%, n=3). A recent infection (intra-abdominal infection, sepsis, or both) was reported in 42.6% (n=20). In 53.2% of cases (n=25), abdominal surgery occurred before PVT. Only one patient (2.1%) acknowledged using oral contraceptives.

**Figure 1:**
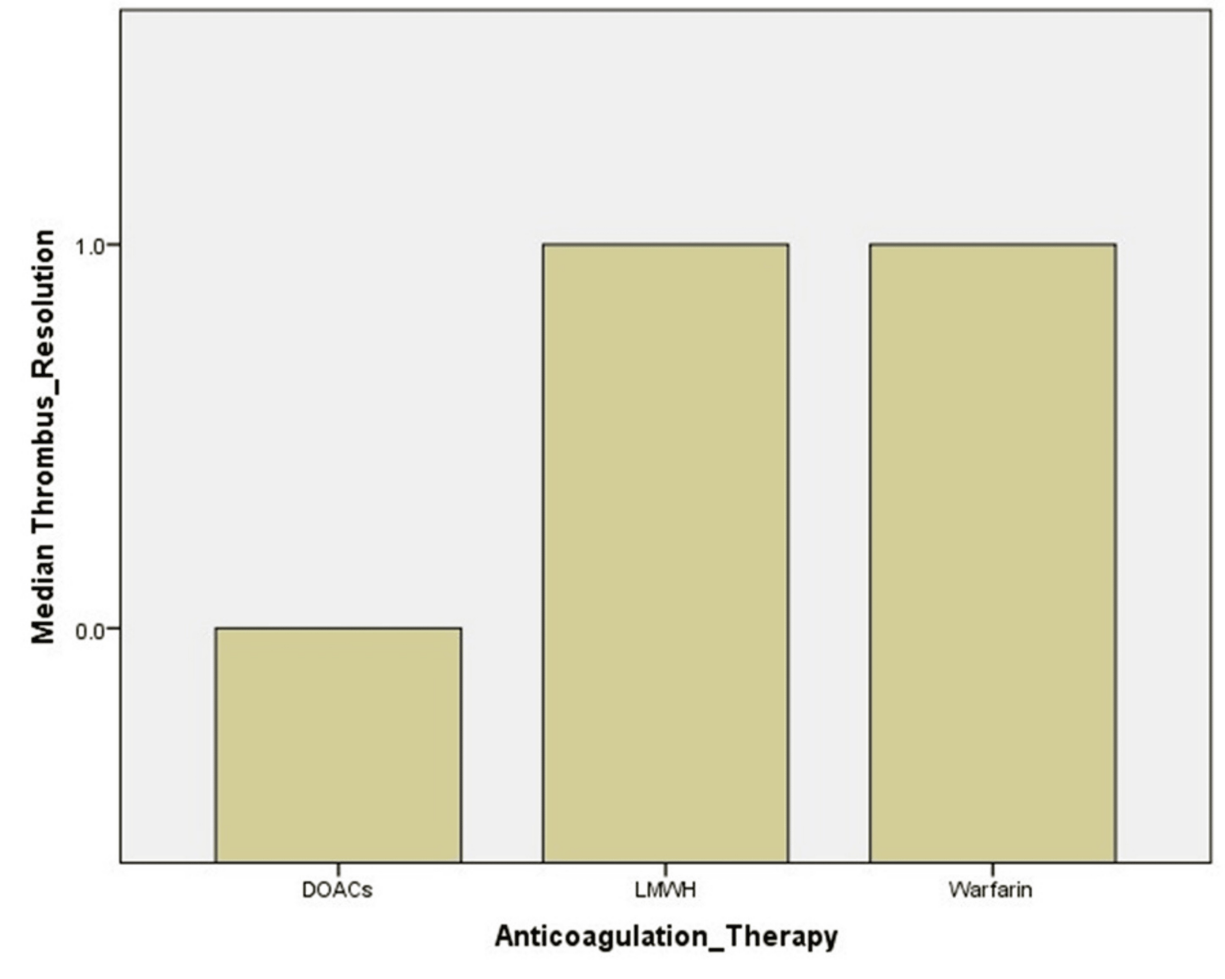
Bar chart of the thrombus resolution by anticoagulation type Data are presented as the median number of patients with thrombus resolution on the y-axis, while the x-axis represents the different types of anticoagulation treatments. The p-value for the association between anticoagulation type and thrombus resolution was 0.450 (Fisher's exact test), with statistical significance defined as p < 0.05. DOACs: Direct oral anticoagulants; LMWH: Low molecular weight heparin

The most common clinical presentation was abdominal pain (72.3%, n=34), followed by ascites (10.6%, n=5) and gastrointestinal (GI) bleeding (4.3%, n=2). A CT of the abdomen was the most common imaging modality (87.2%, n=41). The main portal vein (MPV) alone was the most common location for thrombosis (36.2%, n=17), followed by branches alone (29.8%, n=14) and the MPV with superior mesenteric vein (SMV) involvement (17.0%, n=8). Anticoagulation therapy consisted of LMWH (53.2%, n=25), DOACs (38.3%, n=18), and warfarin (8.5%, n=4). In 29.8% (n=14), the thrombus was completely resolved; in 40.4% (n=19), there was no resolution; and in 29.8% (n=14), the resolution was partial or ambiguous (Figure 1). The median hospital stay lasted seven days (IQR 6-10, range 0-29 days). Portal HTN symptoms (splenomegaly, varices, or ascites) occurred in 17.0% (n=8) of cases, as did bowel ischemia in 10.6% (n=5). The death rates at 30 and 90 days were 14.9% (n=7) and 17.0% (n=8), respectively. During the study period, 95.7% of all hospitalized patients with suspected non-cirrhotic PVT were radiologically confirmed (45/47). Two patients (4.3%) showed negative imaging findings for PVT; they were omitted from subsequent risk factor and outcome analyses.

The type of anticoagulant medication had no statistically significant association with thrombus resolution (Fisher's exact p = 0.450). However, descriptive trends revealed that complete resolution occurred in 36.0% (9/25) of patients getting LMWH, 16.7% (3/18) of patients receiving DOACs, and 50.0% (2/4) of patients using warfarin. There was no resolution found in 32.0% (8/25) of LMWH patients, 55.6% (10/18) of DOAC patients, and 25.0% (1/4) of warfarin patients (Table 2, Figure 1). The median hospital stay was not substantially different between anticoagulation types (Kruskal-Wallis chi-square p = 0.753). Ages were likewise comparable between anticoagulation groups (one-way ANOVA, p = 0.819). When thrombus resolution was divided into two categories (complete vs. no/partial), the correlation remained non-significant (Fisher's exact p = 0.450). An exploratory investigation of DOACs against LMWH found no difference in resolution rates (16.7% vs. 36.0%, p = 0.175).

**Table 2:** Effect of anticoagulation treatment type on the thrombus resolution *Fisher's exact test. All data are represented as a frequency in the form of n (%). Statistical significance is defined as a p-value < 0.05. LMWH: Low molecular weight heparin; DOACs: Direct oral anticoagulants

Anticoagulation type	No resolution (n=19)	Complete resolution (n=14)	Partial/uncertain (n=14)	Total	df	p-value*
LMWH (n=25)	8 (32.0%)	9 (36.0%)	8 (32.0%)	25	4	0.450
DOACs (n=18)	10 (55.6%)	3 (16.7%)	5 (27.8%)	18		
Warfarin (n=4)	1 (25.0%)	2 (50.0%)	1 (25.0%)	4		

Bowel ischemia occurred in five patients (10.6%). There was a strong link between intestinal ischemia and recent infection (Fisher's exact p = 0.016). Specifically, 40.0% (2/5) of patients with intestinal ischemia had both intra-abdominal infection and sepsis, 40.0% (2/5) had only intra-abdominal infection, and 20.0% (1/5) had no infection. Among 42 patients without intestinal ischemia, 96.3% (26/27) had no infection, 87.5% (14/16) had intra-abdominal infection alone, 100% (2/2) had sepsis alone, and none had both (Table 3). Bowel ischemia had no significant relationship with thrombus site (Fisher's exact p = 0.560), malignancy (p = 0.117), prothrombotic condition (p = 0.118), age group (p = 0.632), BMI group (p = 0.619), or anticoagulation type (p = 0.852). Patients with intestinal ischemia had a longer median hospital stay than those without (13 days (IQR 10-29) vs. seven days (IQR 4-10)), although the difference was not statistically significant (Mann-Whitney U = 7.5, p = 0.065).

**Table 3:** Correlation between bowel ischemia and recent infection *Fisher's exact test. All data are represented as a frequency in the form of n (%). Statistical significance is defined as a p-value < 0.05.

Recent infection	No bowel ischemia (n=42)	Bowel ischemia (n=5)	Total	p-value*
No infection	26 (96.3%)	1 (3.7%)	27	0.016
Intra-abdominal infection alone	14 (87.5%)	2 (12.5%)	16	
Sepsis alone	2 (100%)	0 (0%)	2	
Both infection and sepsis	0 (0%)	2 (100%)	2	

Eight patients (17.0%) had sequelae from portal HTN, including splenomegaly, varices, and ascites. There were no statistically significant relationships between portal HTN and thrombus site (Fisher's exact p = 0.560), recent infection (p = 0.078), malignancy (p = 0.117), prothrombotic condition (p = 0.118), age group (p = 0.632), BMI group (p = 0.619), or anticoagulation type (p = 0.852). There was a trend for malignancy (p = 0.049), but cell counts were low (only four of eight patients with portal HTN developed malignancy), and the confidence intervals were broad.

The 30-day mortality rate was 14.9% (seven of 47). Univariate analysis revealed a significant association between 30-day mortality and recent infection (Fisher's exact p = 0.002), malignancy (p = 0.001), and anticoagulation type (p = 0.031). Non-survivors experienced 42.9% (3/7) intra-abdominal infection, 14.3% (1/7) sepsis, and 28.6% (2/7) infection with sepsis. Malignancy was found in 85.7% (6/7) of non-survivors versus 14.3% (1/7) of survivors. All seven deaths occurred in individuals receiving LMWH (28.0% mortality rate in the LMWH group, 7/25); no deaths occurred in patients on DOACs or warfarin. There was no significant age difference between survivors and non-survivors (mean 48.4 vs. 55.3 years, t-test p = 0.392).

The 90-day mortality rate was 17.0% (8 of 47). The univariate correlations with 90-day mortality are shown in Table 4. For the recent infection (Fisher's exact p = 0.009), among non-survivors, 37.5% (3/8) had an intra-abdominal infection, 12.5% (1/8) had sepsis, and 25.0% (2/8) had both. For the malignancy, Fisher's exact p < 0.001; the presence of hepatobiliary or pancreatic cancer was related to significantly increased mortality: 66.7% (4/6) of patients with hepatobiliary cancer and 66.7% (2/3) with pancreatic cancer died within 90 days, compared to only 2.9% (1/35) of those without cancer. For the anticoagulant type (Fisher's exact p = 0.018). All eight deaths occurred in individuals receiving LMWH (32.0% mortality rate in the LMWH group, 8/25); no deaths occurred in people on DOACs or warfarin. The longer hospitalization (Mann-Whitney U = 84.0, p = 0.041). The median hospital stay among 90-day survivors was six days (IQR 4-10), compared to 14.5 days (IQR 9-27) for non-survivors.

**Table 4:** Univariate associations with 90-day mortality *Univariate linear regression analysis. All data are represented as a frequency in the form of n (%). Statistical significance is defined as a p-value < 0.05. LMWH: Low molecular weight heparin; MPV: Main portal vein

Variable	Survivors (n=39)	Non-survivors (n=8)	Test statistic	p-value*
Age, years	49.1 ± 18.7	51.1 ± 24.2	t = -0.272	0.787
BMI, kg/m²	29.0 (24–35)	30.0 (23–32)	U = 88.5	0.056
Hospital stay, days	6 (4–10)	14.5 (9–27)	U = 84.0	0.041
Malignancy	1 (2.6%)	7 (87.5%)	Fisher's exact	<0.001
Recent infection	14 (35.9%)	6 (75.0%)	Fisher's exact	0.009
LMWH anticoagulation	17 (43.6%)	8 (100%)	Fisher's exact	0.018
MPV thrombus	13 (33.3%)	4 (50.0%)	Fisher's exact	0.423
Prothrombotic disorder	7 (17.9%)	1 (12.5%)	Fisher's exact	1.000

There was no significant age difference between survivors and non-survivors (mean 49.1 vs. 51.1 years, t-test p = 0.787). Non-survivors had a lower BMI than survivors (median 30.0 vs. 29.0, p = 0.056). There was no significant relationship between 90-day mortality and thrombus site (p = 0.327), prothrombotic status (p = 1.000), or BMI category (p = 0.289).

A binary logistic regression model for 30-day and 90-day mortality was attempted (Table 5). The regression model for both variables of mortality failed to converge due to the small number of events (eight deaths) compared to the number of predictors (n=13 variables), resulting in unstable estimates (high standard errors). In both models, only the LOS was found to be significantly linked with death. However, this measure should be treated with caution because it is most likely a proxy marker of disease severity and clinical course rather than a reliable baseline predictor of mortality. There were no significant connections between age, BMI, and LOS (Pearson correlation coefficients: -0.126 to 0.082, all p > 0.05). Similarly, Spearman's rank correlations were not significant.

**Table 5:** Binary logistic regression models of mortality *LOS: Length of hospital stay. Statistical significance is defined as a p-value < 0.05.

Variable		B	S.E.	Wald	df	Sig.	Exp(B)	95% CI for EXP(B)	
								Lower	Upper
30-day mortality	Age	0.038	0.026	2.201	1	0.138	1.039	0.988	1.093
	BMI	-0.161	0.12	1.785	1	0.182	0.852	0.673	1.078
	LOS	0.126	0.056	5.056	1	0.025	1.134	1.016	1.266
	Constant	-0.736	2.919	0.064	1	0.801	0.479		
90-day mortality	Age	0.029	0.027	1.221	1	0.269	1.030	0.977	1.085
	BMI	-0.175	0.124	1.992	1	0.158	0.839	0.658	1.071
	LOS	0.171	0.064	7.180	1	0.007	1.186	1.047	1.345
	Constant	-0.182	3.036	0.004	1	0.952	0.833		

## Discussion

Given its retrospective approach, single-center setting, and small sample size, this study should be seen as a descriptive, hypothesis-generating examination of non-cirrhotic PVT in a Middle Eastern cohort. The current single-center retrospective study on a group of 47 patients admitted to the Armed Forces Hospital, Southern Region, with suspected non-cirrhotic PVT yielded interesting results. First, the proportion of clinically suspected patients with radiographically confirmed PVT reached 95.7%, which demonstrates the high sensitivity of clinical suspicion and imaging techniques. Second, the mean age in the study was 49.4 years, and the cohort consisted predominantly of women (61.7%), including those with obesity (42.6%). Third, the percentage of patients whose thrombus completely resolved was only 29.8%, with similar rates among patients using LMWH (36.0%), DOACs (16.7%), and warfarin (50.0%) (p = 0.448). Fourth, recent infection was significantly associated with bowel ischemia (p = 0.016) and both 30-day and 90-day mortality rates. Fifth, the strongest predictor of death in our population was the presence of an underlying malignancy, which had the highest mortality rate, reaching 87.5%. Sixth, even though all deaths involved LMWH users, this result is probably due to indication bias (i.e., LMWH usage in the sickest patients) rather than any actual variation in the safety or effectiveness of anticoagulants. Seventh, hospital stay correlated positively with the 90-day mortality rate (p = 0.041). Eighth, portal HTN complications occurred in 17% of cases and were not related to any risk factor. Our conclusion of a mean age of 49.4 years is consistent with previous literature. A previous multicenter study was conducted in Mexico City that included 100 Hispanic individuals with non-cirrhotic PVT, and the mean age at diagnosis was 38.5 (18-76) years [7]. According to recent comprehensive research by Gil-Lopez et al., non-cirrhotic PVT primarily affects young persons, resulting in a considerable disease burden in this demographic [7]. Another retrospective cohort study undertaken in Oman studied 24 non-cirrhotic PVT patients with a mean age of 52 years and a male predominance [8]. In a study from Mexico, 25 patients with non-cirrhotic PVT had a mean age of 57 ± 13.2 years and female predominance [9]. The female predominance in our population (61.7%) is consistent with research findings that non-cirrhotic PVT is more common in women, despite some geographic variance [4].

Obesity rates in our sample (42.6%) are variable compared to previously reported. A previous French study showed that overweight (36%) was observed in 82% of patients who had idiopathic PVT, compared to 46% of obese patients [10]. While Abdel-Razik et al. (2021) found that PVT patients had an average BMI of 27.3 ± 3.5 kg/m² [11], the rising global obesity epidemic may be changing this landscape. The greater frequency in our group may reflect overall population trends in the Middle East, where obesity rates are among the highest in the world [12,13], or it could imply that obesity is an underappreciated risk factor for PVT, presumably mediated by a pro-inflammatory and prothrombotic state. Prothrombotic abnormalities were found in 17.0% of patients, which is significantly lower compared to other European research, where it ranges from 30% to 40%. According to the recent Hispanic cohort research conducted by Gil-López et al., thrombophilia was seen in 49% of non-cirrhotic PVT patients; antiphospholipid syndrome (APS) was the most frequent (23%), followed by JAK2 mutation (18%) [7]. The reasons for the low frequency of thrombophilia can be associated with the absence of systematic investigation, which occurred only at the doctor's discretion. The high prevalence of the JAK2 mutation in the population of Western origin contrasts with our results. In a study involving 141 Chinese non-cirrhotic PVT patients, Qi et al. found the JAK2 V617F mutation in 25% of patients under investigation; furthermore, overt MPNs were found in 9% [14]. Interestingly, another study found no instances of factor V G1691A or factor II G20210A mutations in their sample population [15].

The prevalence of malignancy in our group (25.5%) is consistent with previous data. In a Danish cohort study of 1,191 patients with splanchnic vein thrombosis, Søgaard et al. (2015) discovered that splanchnic venous thrombosis (SVT) is a significant indication of occult cancer, with a three-month cancer risk of 8.0% and a standardized incidence ratio (SIR) of 33 (95% CI: 27-40), compared to the general population [16]. The greatest risks were for liver cancer (3.5% risk; SIR = 1805), pancreatic cancer (1.5% risk; SIR = 256), and MPNs (0.7% risk; SIR = 764) [17]. Our cohort's high 90-day mortality rates for hepatobiliary (66.7%) and pancreatic (66.7%) malignancies align with Søgaard et al.'s (2015) finding that SVT is a prognostic factor for poor survival in patients with liver and pancreatic cancers [16]. Similarly, the results of another recently published study investigating the role of tumor thrombus and metastasis in cancer-associated SVT demonstrated that the overall survival rate at 12 months was 39.7%, with tumor thrombus (OR 2.44; 95% CI: 1.32-4.52) and metastatic disease (OR 3.07; 95% CI: 1.63-5.8) emerging as independent predictors of mortality [17]. The strong association between infection and intestinal ischemia (p = 0.016) represents an important finding that has been widely explored in the literature.

The prevalence of intestinal ischemia (2.3%) in hospitalized patients with non-cirrhotic PVT was investigated in a nationwide study carried out by Isaac-Coss et al. [6]. In total, the authors analyzed 25,863 inpatient records for non-cirrhotic PVT and found that the incidence of intestinal ischemia was 2.3%. Interestingly, sepsis was the strongest predictor of intestinal ischemia (aOR 2.11; 95% CI: 1.75-2.55; p < 0.001), followed by age and female sex [6]. Importantly, our findings regarding the presence of infection and sepsis in 40% of patients with intestinal ischemia corroborate the data of the aforementioned population study. According to Isaac-Coss et al., the presence of intestinal ischemia independently predicted ICU admission (aOR 2.22), in-hospital mortality (aOR 2.23), longer duration of stay (+2.63 days), and higher hospitalization expenditures (+$82,442) [6]. While our small sample size precluded multivariate analysis, we found that patients with intestinal ischemia had a statistically longer median hospital stay (13 vs. 7 days, p = 0.065), which is consistent with these findings. Our cohort's complete thrombus resolution rate of 29.8% is within the range of previous studies. The recent thorough review by Gil-Lopez et al. emphasizes that long-term anticoagulation may be appropriate even in the absence of evident inciting factors or underlying thrombophilia, while evidence supporting this approach is sparse [17]. In a Korean study of 78 patients with inflammatory bowel disease with PVT, anticoagulant usage had no significant impact on complete radiographic resolution rates (96.2% overall), implying that positive results may occur even without anticoagulation in some populations [18]. However, the high-resolution rate in that study (96.2%) differs significantly from our findings (29.8%), most likely due to differences in patient groups (IBD-specific vs. general) and the inclusion of chronic PVT patients.

Regarding cancer-associated SVT, Garcia-Villa et al. found that anticoagulant therapy was associated with a significantly higher accumulated incidence of recanalization but also with increased bleeding risk, with no benefits observed for survival or prevention of thrombosis recurrence [17]. This highlights the need for individualized risk-benefit assessment when initiating anticoagulation in PVT patients with malignancy. The lack of a significant difference in thrombus resolution between LMWH, DOACs, and warfarin in our study (p = 0.448) is consistent with recent observational studies. The 2025 European guidelines on splanchnic vein thrombosis management recommend anticoagulation for all patients with acute symptomatic PVT but do not specify a preferred agent, reflecting the lack of head-to-head trial data [19]. The prevalence of portal HTN complications (17.0%) in our group is lower than that found in long-term follow-up studies. A Hispanic cohort of 100 non-cirrhotic PVT patients was tracked for a median of 55 months, and the five-year risk of variceal bleeding was 45% [7]. The lower rate in our study could be attributed to the relatively short follow-up period (90 days), as portal HTN problems usually arise months to years following an acute thrombotic event.

Our 90-day mortality rate was 17.0%, which is similar to previous literature. Our Hispanic cohort study found a four-year survival rate of 97% [7]; however, this may be attributed to a lower number of malignancy-related events. On the other hand, the survival rate at 12 months for the cancer-associated SVT cohort in Garcia-Villa et al.'s study was 39.7% [17], demonstrating the impact of the associated malignancy on the prognosis of these patients. Our statistical relationship between LMWH treatment and death (p = 0.018) can be attributed to indication bias since LMWH treatment favors acutely ill patients, sepsis, and aggressive malignancies. The Hokusai venous thromboembolism cancer study did not find a significant difference in mortality between edoxaban and dalteparin among cancer patients with venous thromboembolism, proving the safety of the medication [20].

This study makes many unique contributions. First, it is the first-ever study at the Armed Forces Hospital from the southern region of Saudi Arabia, giving regional evidence for non-cirrhotic PVT in the Middle East. Second, there is significant evidence showing a relationship between recent infections and bowel ischemia (p = 0.016) and 90-day mortality (p = 0.009). It adds up to recent national-level evidence indicating that sepsis is the most potent predictor of bowel ischemia [21]. Third, there is evidence showing thrombus resolution via LMWH, DOACs, and warfarin in a population with a significant rate of obesity (42.6%). Finally, there is confirmation of malignancy as the most potent mortality predictor (p < 0.001), which is in line with literature demonstrating splanchnic vein thrombosis as both a cancer indicator and a poor prognostic sign [16]. Importantly, this result should not be taken as proof of varying efficacy or safety. Instead, it most likely reflects indication bias because the most critically sick patients, such as those with sepsis, multiorgan failure, and aggressive malignancies, were given preference while using LMWH. Warfarin and DOACs, on the other hand, were usually started in stable, discharged patients. Our data does not support any idea of a protective effect from warfarin or DOACs, which would be a classic error of confounding by indication. No reliable comparison between anticoagulant groups can be done without randomization, multivariable correction (which was not achievable due to limited event numbers), or propensity scoring.

There are some shortcomings worth mentioning. The lack of central adjudication of imaging findings or outcome events introduces potential information bias; the instability of statistical estimates due to the small number of outcome events (only eight deaths) precludes reliable multivariable modeling; the lack of standardized protocols for follow-up imaging timing and interpretation, with imaging performed at the discretion of treating physicians rather than per a uniform schedule; and the inherent selection bias associated with any single-center retrospective design limits generalizability. This study should be viewed as hypothesis-generating rather than final. The observed association between LMWH use and mortality is almost certainly driven by indication bias, and no causal conclusions can be drawn regarding anticoagulant choice and survival. Additionally, since there was no control group, there were no opportunities for identifying independent risk factors. Our use of complete-case analysis, which was required by the retrospective methodology, may have increased bias if missing data were not fully random. However, missingness for primary variables was minimal. Nonetheless, we admit that excluding cases with missing records, even if rare, may diminish statistical power and limit generalizability. Future prospective research should employ techniques to reduce missing data and include multiple imputation or sensitivity analysis to determine robustness.

## Conclusions

Our findings suggest that non-cirrhotic PVT carries a substantial risk of adverse outcomes, particularly among patients with recent infectious events or concomitant malignancy. Malignancy is the most significant predictor of death. Only one-third of patients experience complete thrombus resolution, and there is no significant difference between anticoagulation regimens. While all deaths occurred among LMWH recipients, this finding is almost certainly explained by indication bias and should not be interpreted as evidence favoring any particular anticoagulant. Prolonged hospitalization (more than 10 days) indicates a poor prognosis. Clinicians should keep a close eye out for PVT in high-risk patients, treat infections aggressively, check for cancer, and tailor anticoagulation to the individual patient. Larger prospective studies are urgently required.

## Appendices

Appendix A 

Table 6 features the standardized data collection form used to obtain pertinent information methodically from the hospital's EMRs.

**Table 6:** Non-cirrhotic PVT data abstraction form PVT: Portal vein thrombosis; PV: Portal vein; GI: Gastrointestinal; MPN: Myeloproliferative neoplasm; LMWH: Low molecular weight heparin; DOACs: Direct oral anticoagulants

Section	Variable	Response options
Patient ID	Serial number	
Demographics		
Age	Years	
Age group	Years	□ ≤20 □ 20-40 □ 41-60 □ >60
Gender		□ Female □ Male
BMI	kg/m²	
BMI group		□ Healthy weight (18.5-24.9) □ Overweight (25.0-29.9) □ Obesity (≥30.0)
Smoking		□ Yes □ No
Risk Factors		
Prothrombotic conditions		□ None □ Factor V Leiden □ Antiphospholipid syndrome □ MPN □ Protein C/S deficiency □ Prothrombin mutation □ Mixed □ Other
Malignancy		□ None □ Hepatobiliary □ Pancreatic □ Hematologic □ Other
Recent infection		□ None □ Intra-abdominal infection □ Sepsis □ COVID-19 □ Both
Recent abdominal surgery		□ Yes □ No
Oral contraceptives/hormones		□ Yes □ No
Clinical Presentation		
Symptoms		□ Asymptomatic □ Abdominal pain □ GI bleeding □ Ascites □ Abdominal pain + ascites □ Abdominal pain + GI bleeding □ Other
Diagnostic Findings		
Imaging modality		□ CT abdomen □ MRI abdomen □ Doppler ultrasound
Thrombus location		□ Main PV only □ Branches only □ Main PV + branches □ Main PV + SMV □ Splenic vein □ No PVT □ Mixed
Treatment		
Anticoagulation therapy		□ LMWH □ DOACs □ Warfarin □ None
If DOACs, specify		□ Apixaban □ Rivaroxaban □ Other
Outcomes		
Thrombus resolution		□ Complete □ Partial/uncertain □ No resolution
Bowel ischemia		□ Yes □ No
Portal hypertension		□ Yes (splenomegaly/varices/ascites) □ No
Length of hospital stay	Days	
30-day mortality		□ Yes □ No
90-day mortality		□ Yes □ No
Data abstractor initials: ________ Date of abstraction: ________		
